# Simple Routes to Stable Isotope-Coded Native Glycans

**DOI:** 10.1021/acs.analchem.3c03446

**Published:** 2023-12-28

**Authors:** Johannes Helm, Clemens Grünwald-Gruber, Jonathan Urteil, Martin Pabst, Friedrich Altmann

**Affiliations:** Department of Chemistry, University of Natural Resources and Life Sciences Vienna, Muthgasse 18, 1190 Vienna, Austria

## Abstract

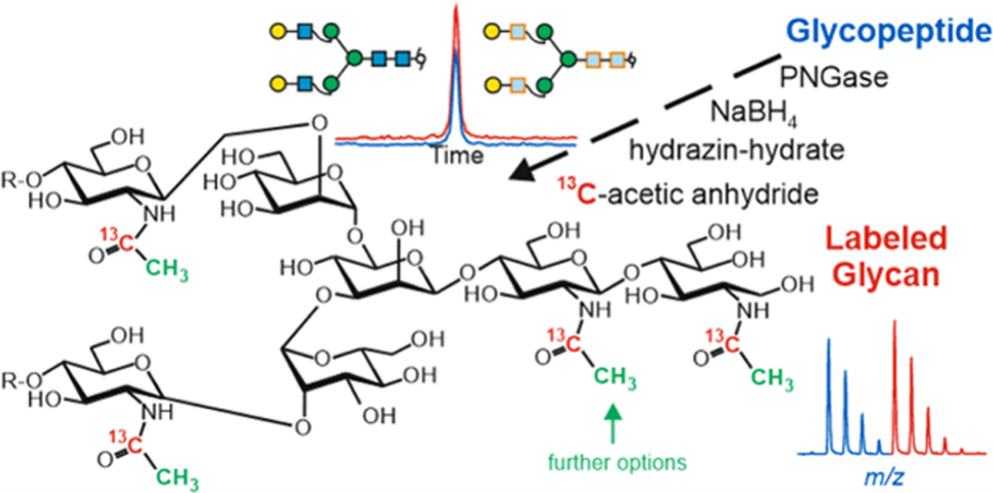

Understanding the biological role of protein-linked glycans requires the reliable identification of glycans. Isomer separation and characterization often entail mass spectrometric detection preceded by high-performance chromatography on porous graphitic carbon. To this end, stable isotope-labeled glycans have emerged as powerful tools for retention time normalization. Hitherto, such standards were obtained by chemoenzymatic or purely enzymatic methods, which introduce, *e.g*., ^13^C-containing *N*-acetyl groups or galactose into native glycans. Glycan release with anhydrous hydrazine opens another route for heavy isotope introduction via concomitant de-*N*-acetylation. Here, we describe that de-*N*-acetylation can also be achieved with hydrazine hydrate, which is a more affordable and less hazardous reagent. Despite the slower reaction rate, complete conversion is achievable in 72 h at 100 °C for glycans with biantennary glycans with or without sialic acids. Shorter incubation times allow for the isolation of intermediate products with a defined degree of free amino groups, facilitating introduction of different numbers of heavy isotopes. Mass encoded glycans obtained by this versatile approach can serve a broad range of applications, *e.g*., as internal standards for isomer-specific studies of *N*-glycans, *O*-glycans, and human milk oligosaccharide by LC–MS on either porous graphitic carbon or—following permethylation—on reversed phase.

## Introduction

Protein-linked glycosylation exerts a wide range of structural and biological functions across all domains of life. In humans, protein glycosylation is the most abundant protein modification, and defects in glycan biosynthesis and metabolism (*a.k.a*. congenital disorders of glycosylation) may cause serious malfunction of different organs.^1^ Moreover, *N*- and *O*-glycans are also useful biomarkers and targets for detection and treatment of other major diseases such as cancer.^2−7^*N*- and *O*-glycans show enormous structural diversity, yet individual structures can confer specific functions. A well-known example is the involvement of sialyl-LeX glycans in inflammatory processes via lectin-based leukocyte homing.^8^ Individual glycan structures can also influence the serum half-life of glycoproteins and therefore the pharmacological efficacy of protein therapeutics.^9^ Current high-throughput methods for glycan analysis, however, determine masses only and separate or identify structural isomers to only a very limited degree. Nevertheless, the occurrence of a large number of isomeric glycan structures has broad biological implications and therefore requires increased attention. Currently, chromatographic separation on porous graphitic carbon (PGC) provides the most promising solution for the detection of isomeric glycan structures.^10−21^ Identification of chromatographic peaks is usually facilitated by negative mode fragmentation,^15,22−25^ while utilization of retention times with the aid of stable isotope-labeled glycan standards (SIL glycans) can substantially contribute to unambiguous assignments.^26−28^ Such SIL glycans can be obtained by chemical synthesis,^26^ by enzymatic incorporation of ^13^C-galactose^28,29^ or by re-*N*-acetylation of glycans following their release by hydrazinolysis.^30^ The latter requires the use of anhydrous hydrazine, which is toxic and explosive and is subject to trading restrictions. Hydrazinolysis can be applied to release *O*-glycans^31,32^ and, under harsher conditions, also for the release of *N*-glycans.^33−35^ However, since the commercialization of peptide-*N*-glycosidases (PNGase), *N*-glycans are commonly released enzymatically.

The detrimental features of anhydrous hydrazine have prompted others to apply aqueous hydrazine for the release of *O*-glycans^36^ and *N*-glycans.^37^ Given the assumption that even traces of water in the release reagent may cause extensive degradation of glycans by peeling at the reducing end, these suggestions are surprising. Indeed, these protocols were hardly employed by the broader research community. Furthermore, reducing *N*-glycans can be obtained via oxidative release^38,39^ or carboxamide rearrangement.^40^ Yet other approaches employ ammonia to overcome alkaline peeling.^41^ Against intuition, reducing *N*-glycans can also be obtained by reductive alkaline release, which—depending on the reaction conditions—also provides species with an amine on C1 of the linking GlcNAc.^42^ It seems, however, that for small- to medium-scale glycan preparations, the application of PNGase F is preferred due to its high specificity and avoidance of side products. While reducing glycans are required for subsequent labeling reactions, underivatized reduced glycans are usually employed for PGC chromatography to simplify the analysis of isomeric structures.

Isotope-labeled glycans serve to normalize retention times, which is a prerequisite for the involvement of retention for isomer identification rationales. This strategy was recently applied to *N*-glycan analysis,^23,28^ and it is to be expected that isotope-labeled glycans will also prove useful for analysis of *O*-glycans and human milk oligosaccharides (HMOs). Backup of MS/MS-based identification by retention times may not yet be standard, but given the enormous number of HMOs and their isomers,^43^ it probably should be both for analysis of underivatized glycans on PGC^44,45^ or permethylated glycans on reversed phase^44,46^ or PGC.^47^ Alternatively, PGC-LC–MS of reductively aminated glycans was recognized as highly useful.^48^

In this work, we demonstrate that de-*N*-acetylation of free, reduced glycans can be performed under moderate conditions using the readily available, cheap, and safe-to-handle hydrazine hydrate. The introduction of stable isotope-labeled acetyl groups can subsequently be achieved using isotope-labeled acetic anhydride. The usefulness and the limitations of this approach to produce isotope-labeled native glycans for the application as (internal) reference standards for LC–MS-based glycomics are described.

## Experimental Section

### Glycans

*N*-glycans from white beans were prepared by pepsin digestion, (glyco)-peptide extraction on a cation exchange resin, and PNGase A treatment, as previously described.^49^*N*-glycans from bovine fibrin (primarily A^4^A^4^ plus some A^3^A^4^, A^4^A^3^, and A^3^A^3^) were isolated as described.^28,50^ Recombinant erythropoietin (EPO), kindly obtained from Polymun AG (Klosterneuburg, Austria), was the source of tri- and tetra-antennary *N*-glycans. *N*-glycans from the pig brain (obtained from a local butcher) were prepared similarly, except that glycan release was accomplished by PNGase F, which was kindly provided by Dr. Lukas Mach (Department of Applied Genetics and Cell Biology, University of Natural Resources and Life Sciences, Vienna). Hybrid-type structures Man4Gn(AF)-bi and Man5GnF^6^bi were isolated from the pig brain by PGC chromatography, as detailed in the Supporting Information. ^13^C_6_-labeled “A^3^A^3^” was prepared enzymatically using ^13^C_6_-UDP-galactose, as detailed in the Supporting Informations.

Mucin-type *O*-glycans were prepared by reductive β-elimination of bovine submaxillary gland mucin as described.^51^ After incubation, the reaction was quenched with a few drops of glacial acetic acid and desalted using Hypercarb cartridges.

Lacto-*N*-tetraose (LNT) and lacto-*N*-neotetraose (LNnT) were purchased from Sigma-Aldrich (Vienna, Austria). Reduction of glycans was carried out with 1% sodium borohydride in 50 mM NaOH at room temperature overnight. The reaction was quenched by the addition of a few drops of glacial acetic acid, and desalting was performed using HyperSep Hypercarb solid-phase extraction cartridges (25 mg; Thermo Scientific, Vienna).^28^

A sample of human milk was kindly provided by Dr. Helmut Mayer (Department of Food Science and Technology, University of Natural Resources and Life Sciences, Vienna). Two hundred microliters of human milk sample was diluted 1:2 with RO water and centrifuged for 20 min at 5000*g*. The solid fat layer on top was discarded, and the liquid phase was further purified with C18 solid-phase extraction cartridges (Strata C18-E, 50 mg, Phenomenex, Torrance, CA). The cartridges were primed with 500 μL of methanol and equilibrated three times with 500 μL of RO water. The sample was applied and was washed with 500 μL of RO water. The flowthrough was collected, subjected to centrifugal evaporation, and reduced as described above.

### Hydrazinolysis

Glycans were dried in screwcap vials by centrifugal evaporation and taken up in 50 μL of hydrazine monohydrate (Alfa Aesar Kandel, Germany). The vials were tightly closed and incubated at different temperatures for different time periods, as described in the manuscript. Following centrifugal evaporation, the de-*N*-acetylated glycans were purified using HyperSep Hypercarb solid-phase extraction cartridges (25 mg) (Thermo Scientific, Vienna).^24^ As an exception, de-*N*-acetylated EPO *N*-glycans were just dried by evaporation, as they could not be eluted from the cartridges. *O*-glycans and individual human milk oligosaccharides were incubated for 72 h at 100 °C.

### Re-*N*-acetylation

Dry de-*N*-acetylated glycans were re-*N*-acetylated by adding 125 μL of precooled 0.1 M sodium bicarbonate and 12 μL of 1,1′-^13^C_2_ acetic anhydride (Cambridge Isotope Laboratories, Tewksbury, MA) or ^2^H_6_-acetic anhydride (Sigma-Aldrich). The reaction mix was incubated at 4 °C for 1.5 h. Then, it was immediately purified using HyperSep Hypercarb solid-phase extraction cartridges (25 mg; Thermo Scientific, Vienna).^24^

### Analysis and Separation of Glycans

Techniques for separation of differently de-*N*-acetylated glycans, *i.e.*, by hydrophilic interaction chromatography (HILIC) with an amide column, and their analyses by MALDI-TOF MS and LC-ESI–MS/MS with a porous graphitic carbon column and either Q-TOF, ion-trap, or orbitrap MS are detailed in the Supporting Informations.

## Results and Discussion

De-*N*-acetylation of glycans was performed using hydrazine hydrate. In conventional hydrazinolysis, degradation of the released, reducing oligosaccharides is minimized by working under anhydrous conditions. Nevertheless, *N*-acetyl groups are quantitatively removed. This side reaction paves the way for stable isotope labeling of the released glycans. We speculated that reduced glycans could be stable in the presence of water while nevertheless being subject to de-*N*-acetylation.

**Figure 1:**
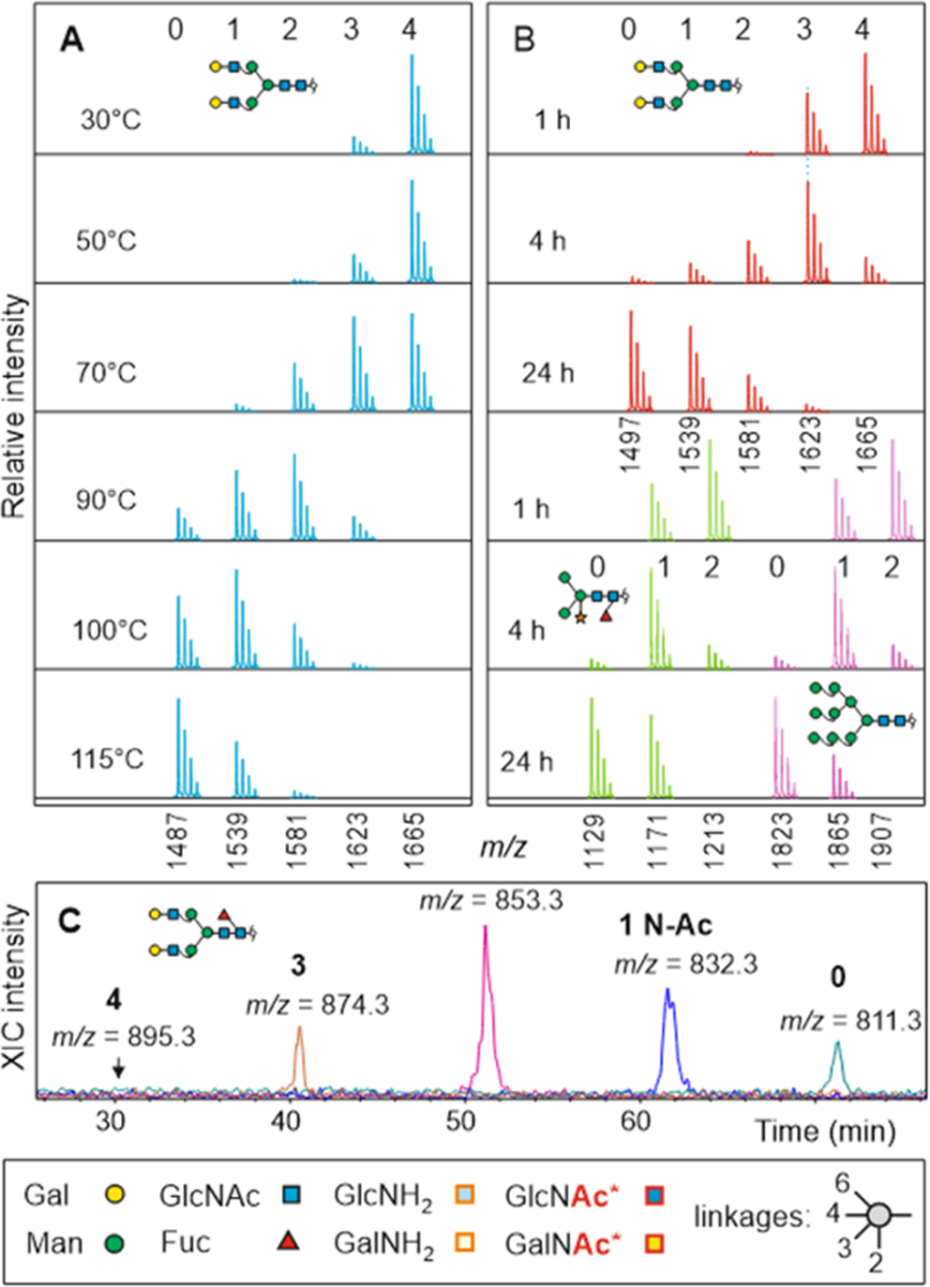
Temperature dependence, time course, and separation of de-*N*-acetylation of *N*-glycans. Isotope pattern schemes represent peak heights of M+Na^+^ ions as measured by MALDI-TOF MS. The cartoons above represent the number of attached *N*-acetyl groups. Nominal *m*/*z* values are listed on the *x*-axis. Panel A shows the degree of de-*N*-acetylation of the biantennary *N*-glycan A^4^A^4^ at different temperatures (30–100 °C) after 24 h of incubation. Panel B depicts a time course of de-*N*-acetylation at 100 °C for the complex *N*-glycan A^4^A^4^ (red), the plant-type structure MMXF^3^ (green), and a high-mannose glycan (purple). Original mass spectra are shown in the Supporting Information (Figures S2 and S3). Panel C shows the separation by amide HILIC HPLC of a 90 °C treatment of A^4^A^4^F^6^. Structure cartoons are drawn in accordance with SFG guidelines^52^ except for de-*N*-acetylated GlcNAc (=glucosamine), GalNAc (=galactosamine), and heavy-isotope-labeled GlcNAc and GalNAc, where Ac* can stand for ^13^C- or ^2^H-acetyl-.

**Figure 2:**
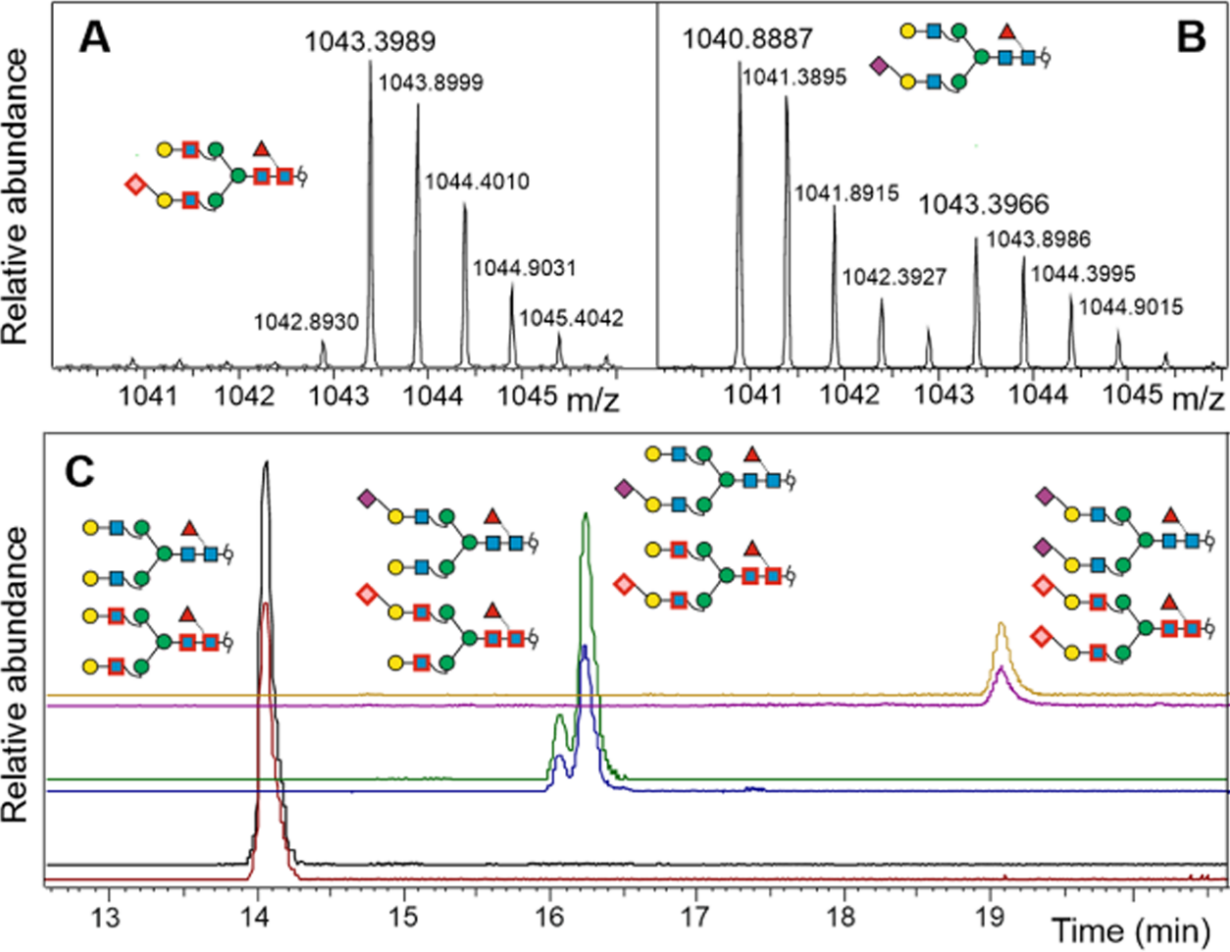
Stable isotope-labeled *N*-glycans analyzed by PGC-LC-ESI orbitrap MS. Panels A and B show the mass spectra of the ^13^C_1_-labeled glycan A^4^Na^6–4^F^6^ from porcine fibrin^50^ alone (A) or in mixture with the native sample (B). For labeling, the glycans had been incubated with hydrazine hydrate for 3 days at 100 °C and subsequently re-*N*-acetylated. The small mass peak before the labeled glycan is derived from isotopic impurity of ^13^C-acetic anhydride and in part from incomplete removal of natural *N*-acetyl groups. Panel C shows the extracted ion chromatograms of the glycans A^4^A^4^F^6^, Na^6–4^A^4^F^6^/A^4^Na^6–4^F^6^, and the disialylated glycan Na^6–4^Na^6–4^F^6^ without (upper traces) and with ^13^C-label (lower traces).

Initial trials revealed that reduced glycans remained intact at high temperatures in aqueous hydrazine hydrate for several hours. The glycans, however, were, to a large degree, smaller by 42 Da (and multiples of) due to de-*N*-acetylation (Figure 1). De-*N*-acetylation occurred with complex-type *N*-glycans with or without core-fucose, with plant *N*-glycans with α1,3-fucose, and with oligomannosidic *N*-glycans in a time- and temperature-dependent manner (Figure 1). Incubation at 100 °C over 72 h consistently resulted in near-to-complete de-*N*-acetylation of diantennary *N*-glycans, as demonstrated in the example of a triplicate analysis (Figure S1). On average, 97–98% of the glycans’ *N*-acetyl group had been removed (Figure S1). A 100% removal, although possible, would anyway be obstructed by the isotopic impurity of the heavy-isotope-containing reagent. The resulting small prepeak does not interfere with the native isotope pattern (Figure 2).

### Influence of the Structural Properties

**Figure 3:**
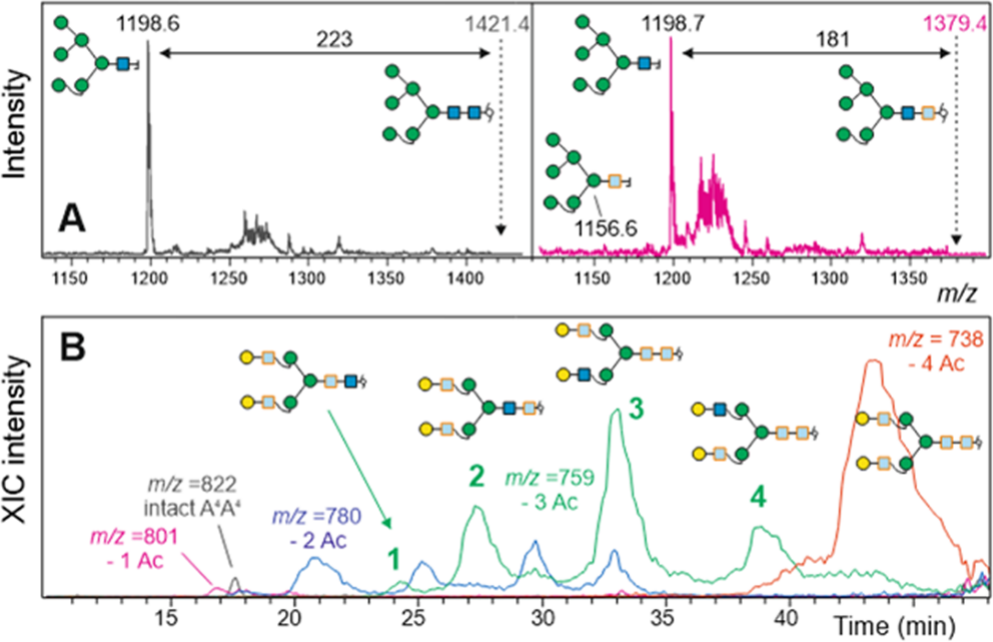
Locating residual *N*-acetyl groups. Panel A depicts laser-induced fragmentation spectra of native and monode-*N*-acetylated Man6, demonstrating that de-*N*-acetylation essentially occurred at the reduced terminal GlcNAc. Panel B shows the result of the analysis of partially de-*N*-acetylated A^4^A^4^*N*-glycans by positive-mode ion-trap PGC-LC–MS/MS. The extracted ion chromatograms show all five stages of de-*N*-acetylation (intact, – 1, – 2, – 3, and −4 *N*-acetyl groups). The numbers give the nominal *m*/*z* of the [M+2H^+^]^2+^ mass peaks.

The MALDI-TOF LIFT MS spectrum of mono-de-*N*-acetylated Man6 showed two B5 ions: one indicative of the emission of complete GlcNAc-itol and the other, more intense one, from its de-*N*-acetylated sibling GlcNH_2_-itol (Figure 3, panel A). Thus, we concluded that the *N*-acetyl group of the reduced terminal GlcNAc residue is particularly susceptible to de-*N*-acetylation. The same observation was made with structure A^4^A^4^, which exhibited a −181 Da peak three times the height of the −223 Da peak (Figure S4). As three internal GlcNAcs compete with the one at the reducing end, this translates into a roughly ten times faster de-*N*-acetylation of the reduced GlcNAc residue.

Application of a mixture of partially de-*N*-acetylated A^4^A^4^ glycans such as those shown in Figure 1 (panel B, 24 h) to PGC-LC–MS showed a complex spectrum with several peaks for most glycoforms (Figure 3). This indicated that the four possible variants of the glycan with only one *N*-acetyl group remaining on the glycan were separated. Now knowing that the reducing GlcNAc de-*N*-acetylated the fastest, negative mode MS/MS allowed to assign each peak to a particular de-*N*-acetylated variant (Figures 2 and S5). Notably, glycans lacking one acetyl group split up into one dominant peak (de-*N*-acetylation of the terminal GlcNAc) and three smaller peaks (one for each of the less accessible GlcNAc residues) (Figure S5).

**Figure 4:**
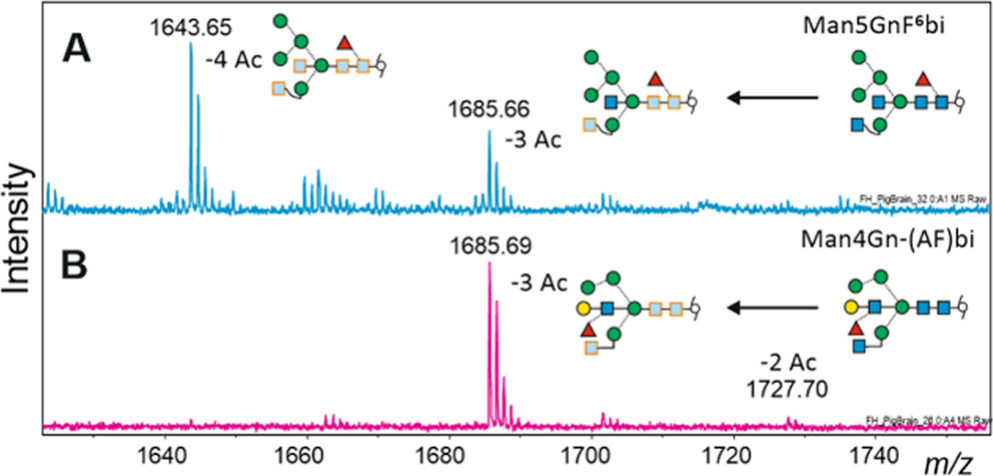
Recalcitrant *N*-acetyl groups. Four *N*-acetyl groups were removed from most of a regular bisected GlcNAc by 72 h incubation with hydrazine hydrate at 100 °C (panel A). The glycan with a substituted bisecting GlcNAc retained one *N*-acetyl group (panel B).

An interesting observation was made with hybrid-type *N*-glycans with bisecting GlcNAc and bisecting Lewis X (as isolated from brain tissue; Figure 4). The structure with two terminal GlcNAc residues readily lost four *N*-acetyl groups. The last step appeared to lag behind that in the biantennary A^4^A^4^, indicating somewhat slower de-*N*-acetylation of the bisecting GlcNAc (data not shown). An almost complete stop before removal of the last *N*-acetyl group was observed with the glycan containing a substituted bisected GlcNAc (Figure 4). This observation might be caused by the nearby substituent in the 3-position. We tested this hypothesis with two experiments: first, with Lewis X determinants on antennal GlcNAc residues and, second, with biantennary glycans bearing type I chains, i.e., β1,3-linked galactose. Three different biantennary glycans—A^4^A^4^, (AF)(AF), and ^13^C-galactose-labeled A^3^A^3^—were mixed and subjected to hydrazinolysis. At different time points, samples were taken and subjected to a PGC-LC–MS analysis. The results shown in Figure S6 confirm that a substituent in the 3-position significantly hinders de-*N*-acetylation.

Interestingly, the XIC traces of variants lacking two *N*-acetyl groups from the different glycans revealed an unexpected detail. Considering that the three internal *N*-acetylglucosamines of A^4^A^4^ are de-*N*-acetylated more slowly than the reduced GlcNAc, one could expect three large peaks with a de-*N*-acetylated first GlcNAc and three small peaks with a still intact first GlcNAc. This consideration predicts in total six isomers in accordance with the binomial coefficient of 4 over 2. In fact, six peaks can be observed in the respective XIC trace of the PGC chromatogram (Figure S6). In striking contrast, both specimens with a 3-position substituent yielded just one peak with two removed *N*-acetyl groups—probably from the GlcNAc residues of the core chitobiose (Figure S6).

### Separation of Partially De-*N*-acetylated Glycans

The obvious approach for separating glycans by their degree of de-*N*-acetylation (=numbers of positive charges) is cation exchange chromatography. However, the lack of a suitable chromophore in the glycans necessitated mass spectrometric detection. The interference by the salt required for elution was minimized by the use of a volatile buffer and extensive vacuum drying of the collected LC fractions. The cumbersome fraction handling and poor performance of the cation exchange separation system (Figure S7) prompted us to investigate other options. Chromatography using porous graphitized carbon of partial hydrazinolysates of A^4^A^4^ showed a highly complex peak pattern with considerable overlapping (Figure 3). ZIC-HILIC was found to separate glycans according to their degrees of de-*N*-acetylation with only little subfractionation based on amino group arrangement (Figure S8). Amide HILIC on a “conventional” HPLC column, however, gave an excellent separation of the different variants of de-*N*-acetylated porcine fibrin glycans^50,53^ and obviously constituted the method of choice for preparative purposes (Figures 1 and S9).

### Introduction of Stable Isotopes into Glycans

De-*N*-acetylated glycans can be labeled with isotopes by re-*N*-acetylation with acetic anhydride, which is available in different ^13^C and ^2^H label versions. To avoid retention time differences between light and heavy glycans caused by the deuterium effect,^54−56^ we directed our efforts to ^13^C-labeled acetic anhydride. In a biantennary *N*-glycan, the re-*N*-acetylation of 4 amino groups with 1,1′-^13^C_2_ acetic anhydride resulted in a mass increase of 4 Da. The heavy glycan coeluted with the light (native) version but did not interfere with its XIC trace (Figure 3). A parallel experiment with the more economic ^2^*H*_6_-labeled acetic anhydride demonstrated that the deuterium-containing glycan—in defiance of our apprehension—also coeluted with the native version (Figure S10).

Finally, we tested the preparation of tri- and tetra-antennary isotope-coded *N*-glycans. A nearly complete de-*N*-acetylation of the tri- and tetra-antennary *N*-glycans was achieved, albeit only following extensive incubation times (Figure S11).

### Application to *O*-glycans

Next, we wanted to investigate whether *O*-glycans, which contain *N*-acetylgalactosamine (GalNAc), can likewise be de-*N*-acetylated by hydrazine for isotope labeling. *O*-glycans with compositions H1N1F1 and H1N2F1, which can be assigned as Fuc(α1–2)Gal(β1–3)GalNAc-ol and Fuc(α1–2)Gal(β1–3)-[GlcNAc(β1–6)]-GalNAc-ol, respectively, were selected as examples.^51^ The *O*-glycans were completely de-*N*-acetylated within 72 h at 100 °C despite the substituent in the 3-position of GalNAc. The accelerated reaction of a reduced terminal amino sugar, as already observed with *N*-glycans, appears to offset the slower pace of de-*N*-acetylation of 3-position-substituted amino sugars.

**Figure 5:**
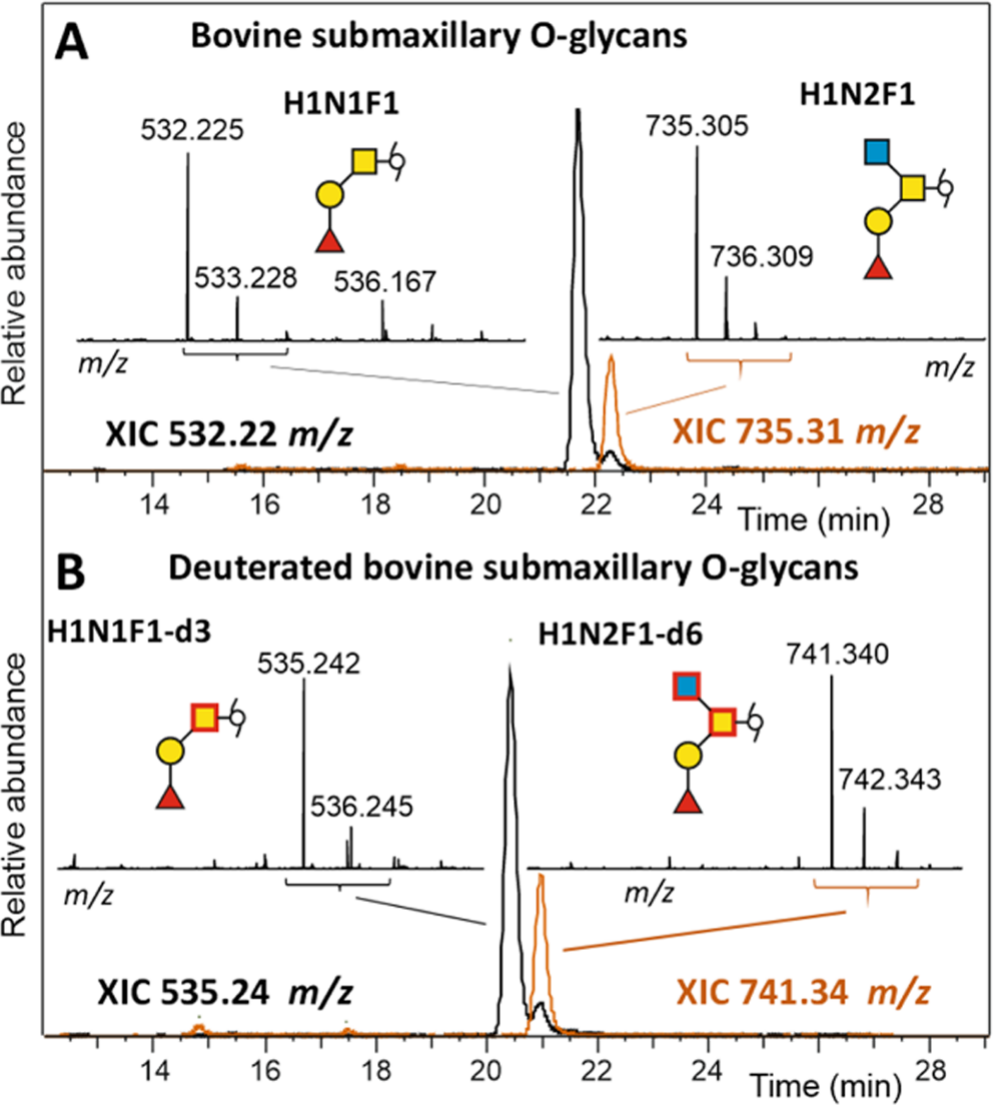
Native and stable isotope-labeled *O*-glycans in separate chromatographic runs. Panel A shows the PGC chromatograms and the mass spectrum of native bovine submaxillary *O*-glycans with compositions H1N1F1 (black line) and H1N2F1 (orange line). Panel B shows the PGC chromatograms and the mass spectrum of isotope-labeled (deuterated) bovine submaxillary *O*-glycans with compositions H1N1F1 (black line) and H1N2F1 (orange line). The fragment spectra of the native and the isotope-labeled H1N1F1 *O*-glycan are shown in Figure S12. The different retention times of the two runs emphasize the importance of the use of internal standards.

Deuterated acetic anhydride was subsequently used to introduce ^2^H_3_-labeled acetyl groups, which—as expected—increased the mass of the H1N1F1 structure by 3 Da and that of H1N2F1 by 6 Da (Figure 5). The retention time shift seen in the two runs emphasizes the need for retention time normalization with the help of isotope-tagged internal standards. The fragment spectra confirm that the mass increments of the respective structures are derived from isotope-labeled HexNAc residues (Figure S12).

### Application to HMOs

**Figure 6:**
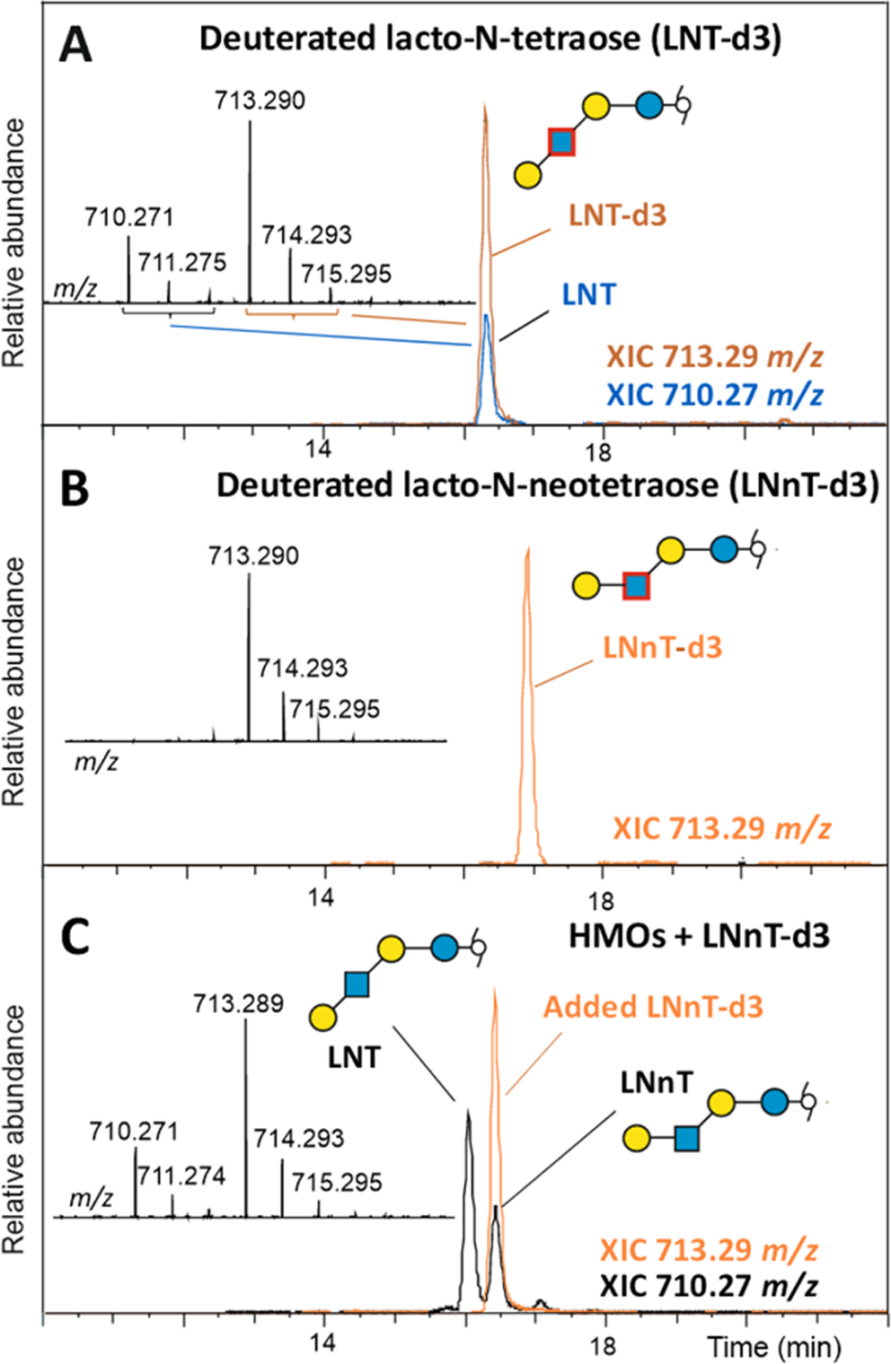
Stable isotope-labeled human milk oligosaccharides. Panels A and B show the PGC chromatograms and mass spectra of deuterium-labeled lacto-*N*-tetraose (LNT-d3) and lacto-*N*-neotetraose (LNnT-d3), respectively. Panel C shows the PGC chromatogram and the mass spectrum of heavy-isotope-labeled LNnT spiked into HMOs extracted from human milk. Note that the chosen HMO fraction may not represent the usually observed natural ratio of LNT and LNnT.

Human milk oligosaccharides (HMOs) are a frequently analyzed large group of glycans, of which many contain *N*-acetylhexosamines and should thus be accessible to isotope labeling by the herein-described approach. To fathom this possibility, we de- and re-*N*-acetylated lacto-*N*-tetraose (LNT) and lacto-*N*-neotetraose (LNnT). This experiment confirmed the observation that a substituent in the 3-position significantly hinders de-*N*-acetylation. LNnT, which possesses a type 2 chain (β1,4-linked galactose to GlcNAc), was completely de-*N*-acetylated after 72 h (Figure 6B), whereas LNT, which possesses a type 1 chain (β1,3-linked galactose to GlcNAc), was de-*N*-acetylated to only about 65% (Figure 6A). Application of the generated isotope-labeled LNnT to human milk oligosaccharides unambiguously identified the smaller and later eluting peak as being LNnT. This observation is in line with Eussen et al., who showed that LNnT is present to a lower extent compared to LNT in human milk.^57^ Obviously, isotope-labeled HMO glycans could assist in qualitative and quantitative analyses of HMOs.

## Conclusions

Hydrazine hydrate opens a convenient solution for the preparation of de-*N*-acetylated *N*-glycans, *O*-glycans, and other oligosaccharides with or without sialic acids. Hydrazine hydrate overcomes the efforts and safety concerns associated with shipping and handling of anhydrous hydrazine and the efforts and costs of working with isotope-labeled nucleotide sugars. Two factors influencing the speed of reaction were identified. Reduced terminal *N*-acetylhexosamines are particularly sensitive to hydrazine hydrate, whereas a 3-position substituent drastically slows down de-*N*-acetylation. A way to accelerate the reaction could be the addition of hydrazonium bromide, as reported in a recent paper dealing with the mechanism of amide cleavage by hydrazine.^58^ The possibility of choosing different mass increments expands the toolbox for isomer-specific “deep” glycomics. While this high-end application requires chromatographic separation, useful preparations of internal standards can also be provided without laborious downstream processing, as shown in Figures 4, 5, and 6.

Application options for isotope-labeled *N*-glycans produced this way may well include glycopeptides. Isotope-encoded glycopeptides were recently introduced as a means for the targeted quantitation of IgG.^59^ The procedure entailed solid-phase synthesis of a glycopeptide containing ^13^C-labeled GlcNAc and transfer of glycan oxazoline with the help of mutated endoglycosidase F3. Endoglycosidase F1 is able to cleave reduced oligomannosidic *N*-glycans.^60^ Endoglycosidases F2, F2, S, and S2, which are able to act on complex-type *N*-glycans, likewise belong to GH family 18 and act via the conserved active site residues DxE.^61−64^ Therefore, it appears highly likely that the isotope-labeled glycans generated as described in this article can serve to build isotope-encoded glycopeptides from exclusively natural substrates.

## Supporting Information Available

Reproducibility of de-*N*-acetylation with hydrazine hydrate; de-*N*-acetylation of the diantennary *N*-glycan A^4^A^4^ by hydrazine hydrate at different temperatures; time course of de-*N*-acetylation by hydrazine hydrate at 100 °C; MALDI-LIFT MS of A^4^A^4^ with one *N*-acetyl group removed; negative mode CID spectra of a biantennary glycan with one remaining GlcNAc; influence of 3-substitution on susceptibility to hydrazine hydrate; separation of de-*N*-acetylation variants by cation exchange chromatography; separation of various de-*N*-acetylated glycans by HILIC; coelution of native and of deuterium-labeled glycans on PGC; and isotope coding of tri- and tetra-antennary *N*-glycans (PDF)
