# Supplementary material for: Simple Routes to Stable Isotope-Coded Native Glycans
Source: Anal Chem. 2023 Dec 28;96(1):163–9. doi: 10.1021/acs.analchem.3c03446 (PMC10782419; doi:10.1021/acs.analchem.3c03446)
Supplement: Supplementary file 1 — ac3c03446_si_001.pdf [file ac3c03446_si_001.pdf]

SUPPORTING INFORMATION

for

A simple route to stable isotope coded native glycans.

Johannes Helm, Clemens Grünwald-Gruber[§], Jonathan Urteil[$], Martin Pabst[&], Friedrich Altmann*

Department of Chemistry, University of Natural Resources and Life Sciences Vienna, Muthgasse 18, 1190 Vienna, Austria.

* Email: friedrich.altmann@boku.ac.at

Present addresses:

[§]C.G.-G.: Core Facility, University of Natural Resources and Life Sciences, Muthgasse 11, 1190 Vienna, Austria

[$]J.U.: Biomay AG, Ada-Lovelace-Straße 2, 1220 Wien, Austria

[&]M.P.: Department of Biotechnology, Delft University of Technology, Delft, The Netherlands

Content:

Page	
2	Supporting methods
4	Supporting figures
13	Supporting references

Supporting methods:

N-glycan preparations. N-glycans from white beans were prepared by pepsin digestion, (glyco)-peptide extraction on a cation exchange resin and PNGase A treatment as previously described [1]. N-glycans from pig brain (obtained from a local butcher) were similarly prepared except that glycan release was accomplished by PNGase F, which was kindly provided by Dr. Lukas Mach (Department of Applied Genetics and Cell Biology, University of Natural Resources and Life Sciences, Vienna). N-glycans from bovine fibrin were isolated as described [2, 3]. N-glycans from recombinant erythropoietin (EPO) were available from a previous study [4]. Reduction of glycans was carried out with 1 % sodium borohydride in 50 mM NaOH at room temperature overnight. The reaction was quenched by the addition of a few drops of glacial acetic acid and desalting was performed using HyperSep Hypercarb solid-phase extraction cartridges (25 mg; Thermo Scientific, Vienna).

PGC-LC fractionation. The isolation of the hybrid-type structures Man4Gn-(AF)bi and Man5GnF^{6}bi from the pig brain N-glycome [2, 5] was carried out by fractionation via PGC-chromatography on a Hypercarb-column (150 × 4.6 mm, 5 µm particle size, Thermo Scientific, Vienna, Austria). Buffer A was 80 mM ammonium formate pH 3.0 and buffer B was 80 % acetonitrile in buffer A. The column was held at 30°C and the flowrate was 1 mL/min. The gradient was 0 – 5 min 6 % B, from 5 – 30 min 6 – 30 % B, from 30 – 44 min 30 – 65 % B followed by 10 min equilibration at 100 % A. Fractions were subjected analyzed for the glycans of interest by MALDI-TOF-MS.

Cation exchange fractionation. Separation of deacetylated glycans isomers with different numbers of acetyl groups by cation exchange was performed using a 1 ml Econo-Pac High S cation exchange cartridge (Bio-Rad, Hercules, CA) on an ÄKTA protein purifier (Thermo Scientific, Vienna). Dry glycans were diluted in 100 µl of 50 mM ammonium acetate solution at pH 3.5 and loaded onto the cartridge at a flow rate of 0.5 mL/min. Bound glycans were eluted with a gradient to 100% 2 M ammonium acetate at pH 3.5 in 30 min. Eluted glycans were collected in fractions of 0.5 mL. For desalting, the fractions were subjected to multiple cycles of drying in a rotary evacuator and re-dissolving in water.

ZIC-HILIC fractionation of de-*N*-acetylated glycans. The isolation of the differentially de-*N*-acetylated glycans was carried out by ZIC-HILIC chromatography. The de-*N*-acetylated N-glycans were taken up in 50 uL water and injected onto a Sequant ZIC-HILIC column (150 mm × 1 mm, 3 µm particle size, Merck, Darmstadt, Germany). Buffer A was 80 mM ammonium formate pH 3.0 and buffer B was 80 % acetonitrile in buffer A. The column was held at 40°C and the flowrate was 0.150 mL/min. The gradient was 0 – 2 min 100 % B, from 2 – 26 min 100 – 10 % B followed by 10 min equilibration at 100 % B. The fraction size was 0.150 mL. The fractions were subjected to centrifugal evaporation, taken up in water and analyzed by MALDI-TOF-MS (see below). Alternatively, detection was performed by mass spectrometry on a ion trap (amaZon speed ETD; Bruker, Bremen, Germany) as detailed in below in a separate chapter.

Amide-HILIC. Assuming that this type of HILIC phase would give similar results as ZIC-HILIC we initially hesitated to connect the "conventional" format TSK-Amide80 column (4.6x250 mm, 5 µm; Tosoh Bioscience, Griesheim, Germany). A partially de-*N*-acetylated sample of N-glycans from porcine fibrin essentially containing sialylated $A^4A^4F^6$ was applied to the column operated at 40°C with the eluents described for the ZIC-HILIC experiment. A glycan sample representing approximately 2 mg of fibrin was applied at 95% solvent. From minutes 2 to 6, solvent B content was lowered to 75 % followed by a further decrease to 40 % over 70 min at a flow rate of 0.5 mL/min.

Preparation of $^{13}C_6$-UDP-galactose. UDP-$^{13}C_6$-galactose was prepared by incubation of $^{13}C_6$-galactose (Cambridge Isotope Laboratories, Tewksbury, MA, USA) with galactokinase (Sigma-Aldrich, Vienna, Austria). The resulting Galactose-1-phosphate was converted to the nucleotide sugar in the presence of UDP-glucose by human galactose-1-phosphate uridylyltransferase, which was recombinantly expressed in *Escherichia coli* BL21 and purified via its His6-tag. The UDP-$^{13}C_6$-galactose was finally purified by PGC chromatography with a slightly alkaline buffer as described [6].

Biosynthesis of N-glycan structures. $^{13}C_6$-labeled "A^3A^3" was prepared as previously described [2]. In short, "A^4A^4", prepared from bovine fibrin, was incubated over night at 37°C with β-galactosidase from

Aspergillus oryzae (Sigma Aldrich, Vienna, Austria) in 0.1 M phosphate citrate buffer pH 5.0. The reaction mixture was purified using HyperSep Hypercarb solid-phase extraction cartridges (25 mg) (Thermo Scientific, Vienna). The degalactosylated glycan "GnGn" was furthermore β1,3-galactosylated with recombinantly expressed human β1,3-galactosyltransferase in a total volume of 50 µL in 25 mM Tris/HCl pH 7.4 + 100 mM NaCl supplemented with 1 mM $^{13}C_6$-UDP-galactose and 2 mM $MnCl_2$. The reaction mixture was purified using HyperSep Hypercarb solid-phase extraction cartridges (25 mg) (Thermo Scientific, Vienna).

The doubly α1,3 antenna fucosylated structure "(AF)(AF)" was prepared by subjecting the glycan "A^4A^4" to fucosylation with recombinantly expressed fucosyltransferase IV (FucT-IV) as previously described [2]. In short, "A^4A^4" was incubated over night at 37°C with recombinantly expressed human fucosyltransferase IV in 50 µL in 25 mM Tris/HCl pH 7.4 + 100 mM NaCl supplemented with 1 mM GDP-fucose and 10 mM MnCl2. The reaction mixture was purified using HyperSep Hypercarb solid-phase extraction cartridges (25 mg) (Thermo Scientific, Vienna).

MALDI-TOF MS. Diluted glycan preparations were spotted onto a MALDI target plate and dried. A 2% solution of 2,5-dihydroxybenzoic acid in 50% acetonitrile was added and the droplets were quickly dried in a vacuum desiccator to obtain small uniform crystals. Spectra were acquired with an Autoflex MALDI (Bruker, www.bruker.com) in positive reflectron mode. All spectra were re-calibrated using oligomannosidic glycans as internal standards. Fragment spectra were obtained by laser induced fragmentation in LIFT mode.

PGC-LC-Ion trap-MS. Purified samples were loaded on a PGC-column (100 mm × 0.32 mm, 5 µm particle size, Thermo Scientific, Waltham, MA, USA) with 10 mM ammonium bicarbonate as the aqueous solvent A and 80 % acetonitrile in solvent A as solvent B. The gradient was as followed: 0 – 4.5 min 1 % B, from 4.5 – 5.5 min 9 % B, from 5.5 – 29 min 9 - 20 % B, from 29 – 41.5 min 20 - 35 % B, from 41.5 – 43 min 35.0 – 68.8 % B, followed by an equilibration period at 1 % B from 43 – 50 min. The flow rate was 6 µL/min. Detection was performed with a standard ESI source directly linked to the Thermo Ultimate 3000 UPLC system. All samples were measured with an ion trap instrument (amaZon speed ETD; Bruker, Bremen, Germany) in positive mode or in negative mode. MS-scans were recorded from 400 – 1600 m/z.

The negative mode measurements were carried out with the ICC target set to 100000, maximum accumulation time set to 250 ms and a scan range from 600 – 1500 m/z. The scan mode was set to enhanced resolution and the SPS (Smart Parameter Settings) were set to target mass 950 m/z, compound stability 100% and trap drive level 100%. For MS/MS, the number of precursor ions was set to 3 with active exclusion (exclusion after 1 spectrum and release after 0.22 min). The absolute threshold was set to 10000 and the inclusion list was set from 700 – 1200 m/z. The scan mode for MS/MS was set to enhanced resolution with the ICC target set to 200,000 and maximum accumulation time of 100 ms with isolation width set to 2 m/z. Smart frag was enabled in enhanced mode (start amplitude: 150%, end amplitude 200% and fragmentation time 35 ms). PAN was enabled with Low CID CutOff at 17% and time stretch at 250%. FxD was enabled with activation at 100%, decay at 100% and tickle level at 100%.

The positive mode measurements were carried out with the ICC target set to 165,000, the maximum accumulation time set to 200 ms and a scan range from 600 – 1500 m/z. The scan mode was set to enhanced resolution and the SPS (Smart Parameter Settings) were set to target mass 850 m/z, compound stability 100 % and trap drive level 100%. For MS/MS, the number of precursor ions was set to 3 with active exclusion (exclusion after 1 spectrum and release after 0.25 ms). The absolute threshold was set to 20,000 and the inclusion list to 700 – 1200 m/z. The scan mode for MS/MS was set to enhanced resolution with the ICC target set to 200,000, the maximum accumulation time to 55 ms and the isolation width to 3 m/z. Smart Frag was enabled in enhanced mode (start amplitude 80%, end amplitude 120% and fragmentation time 20 ms). PAN and FxD was disabled for positive mode MS/MS. Standard source settings (capillary voltage 4.5 kV, nebulizer gas pressure 0.5 bar, drying gas 5 L/min, 200 °C) were used. Instrument tuning was optimized for a low mass range (around 1500-2000 Da molecules). MS/MS was carried out in data- dependent acquisition mode (switching to MS/MS mode for eluted peaks). Data interpretation was done with DataAnalysis 4.0 (Bruker, Bremen, Germany).

PGC-LC-Orbitrap-MS. LC-MS analysis was performed on a Dionex Ultimate 3000 UHPLC system coupled to an Orbitrap Exploris 480 Mass Spectrometer (Thermo Scientific). The purified glycans were loaded on a Hypercarb column (100 mm × 0.32 mm, 5 μm particle size, Thermo Scientific, Waltham, MA, USA) with 80 mM ammonium formate as the aqueous solvent A and 80 % acetonitrile in 80 mM ammonium formate as solvent B. The gradient was as follows: 0 – 4.5 min 1 % B, from 4.5 – 5.5 min 1 – 9 % B, from 5.5 – 30 min 9 - 20 % B, from 30 – 41.5 min 20 - 35 % B, from 41.5 – 45 min 35 – 65 % B, followed by an equilibration period at 1 % B from 45–55 min. The flowrate was 6 μL/min. MS analysis was performed in data-dependent acquisition (DDA) mode with positive polarity from 500 – 1500 m/z using the following parameters: resolution was set to 120,000 with a normalized AGC target of 300%. The 10 most abundant precursors (charge states 2-6) within an isolation window of 1.4 m/z were selected for fragmentation. Dynamic exclusion was set at 20 s (n=1) with a mass tolerance of ± 10 ppm. Normalized collsion energy (NCE) for HCD was set to 20, 25 and 30% and the precursor intensity threshold was set to 10000. MS/MS spectra were recorded with a resolution of 15,000, using a normalized AGC target of 100% and a maximum accumulation time of 100 ms.

Supporting figures:

Figure S1. Reproducibility of de-*N*-acetylation with hydrazine hydrate. N-glycans obtained by enzymatic deglycosylation of a pepsin digest of porcine fibrin. The incubation at acidic pH removed most but not all sialic acids. The MALDI-TOF spectra of 1 : 50 dilutions of the samples before (panel **A**) and after 2 (panel **B**) and 3 days (panel **C**) of incubation with hydrazine hydrate at 100°C show highly similar results in the parallel samples with no degradation. The sodium salts of Neu5Ac and Neu5NH_2 appeared with mass increments of 313 and 271 mass units. The percentages in panel **C** are the peak heights relative to maximum. This amount of remaining *N*-acetyl groups translates into a removal of more than 97 % of all acetyl groups.

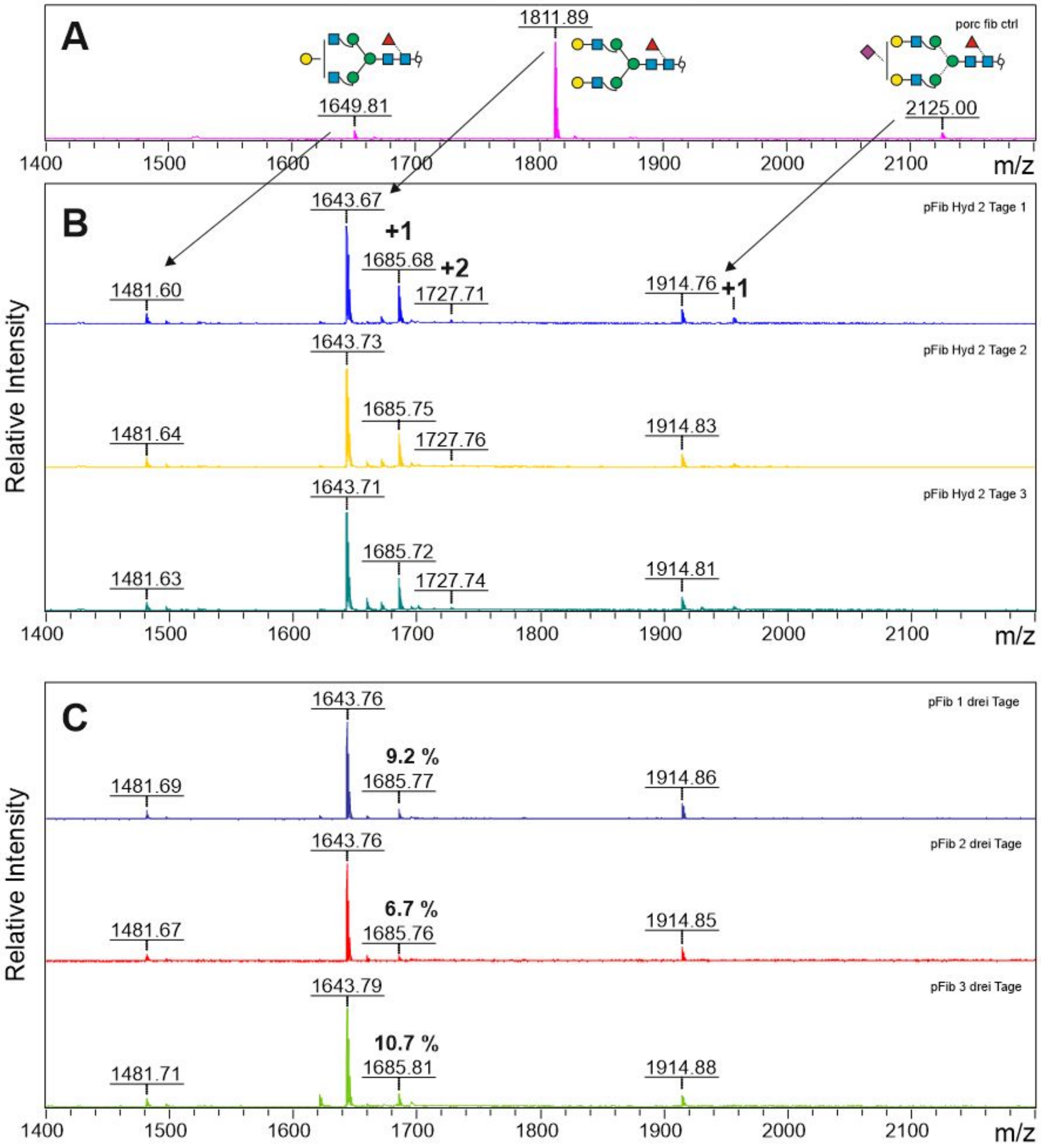

Figure S2. De-*N*-acetylation of the diantennary N-glycan A⁴A⁴ by hydrazine hydrate at different temperatures. Results were monitored by MALDI-TOF MS after a 24 h incubation and subsequent evaporation of the reagent. **Panel A** shows the spectra for the individual temperatures. These results are the basis for the condensed depiction in Figure 1 of the main text. **Panel B** exemplifies the – low - degree of collateral destruction of the substrate.

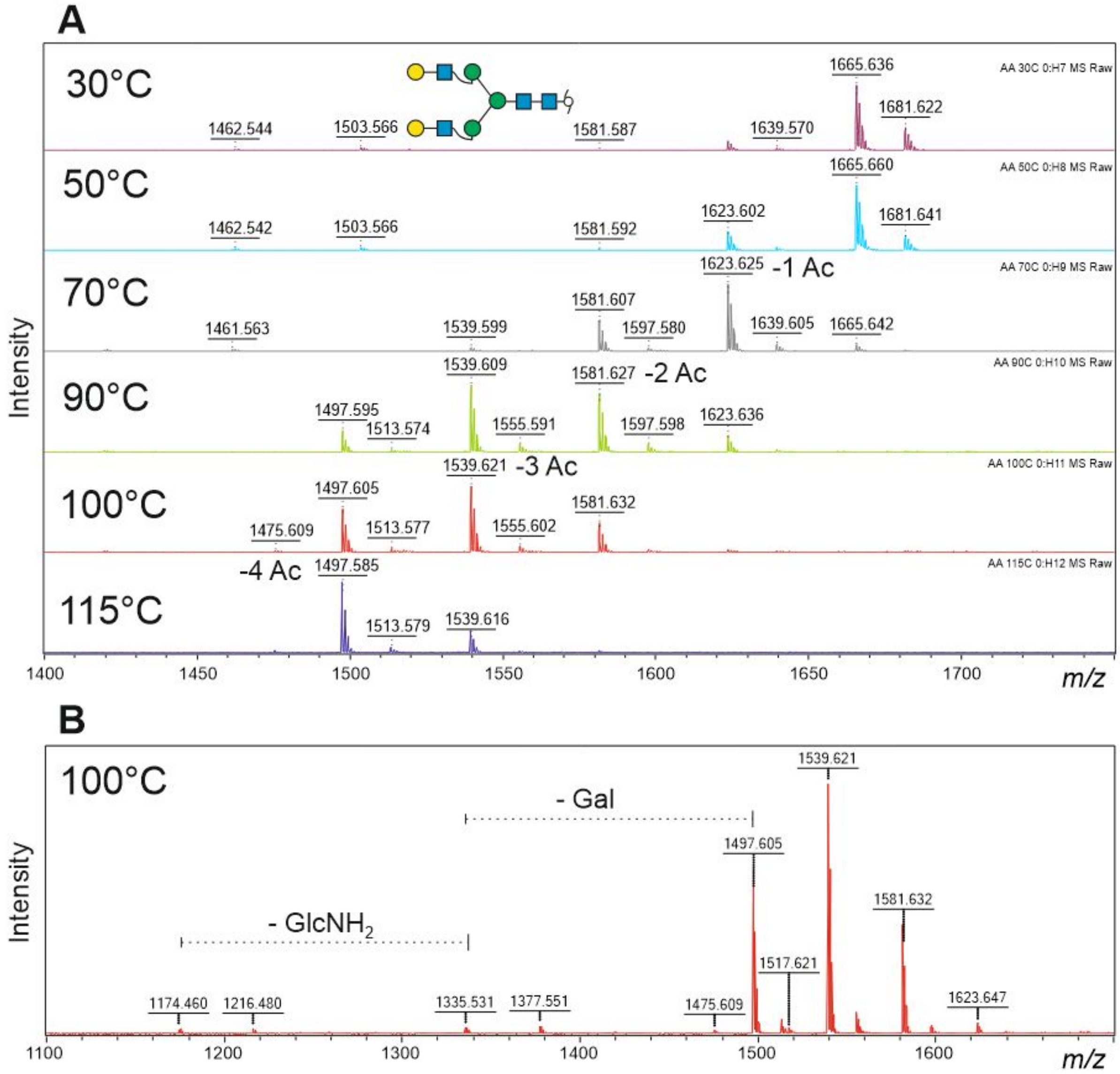

Figure S3. Time course of de-*N*-acetylation by hydrazine hydrate at 100 °C. Biantennary N-glycans (A^4A^4) and a mixture of white bean N-glycans containing oligomannosidic and plant-type complex N-glycans were incubated for the times indicated.

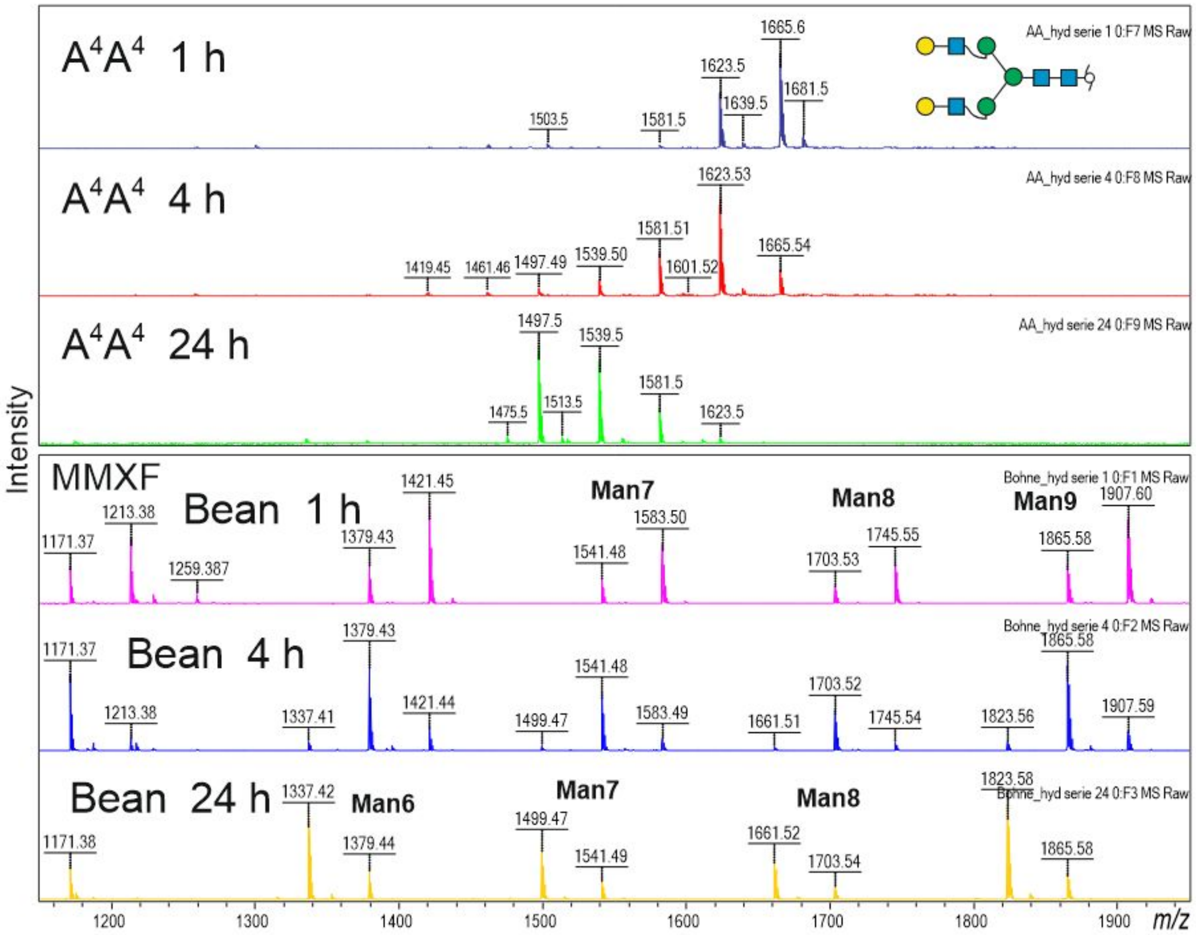

Figure S4. MALDI-LIFT MS of A^4A^4 with one *N*-acetyl group removed. In the LIFT spectrum of precursor 1623.5 (-1 N-acetyl group), the peak at m/z 1442.6 arises from de-*N*-acetylated reducing GlcNAc (actually then $GlcNH_2$). m/z = 1400.7 comprises the three isomers with an internal $GlcNH_2$. The peak height ratio points at the much faster reaction of the reducing end GlcNAc.

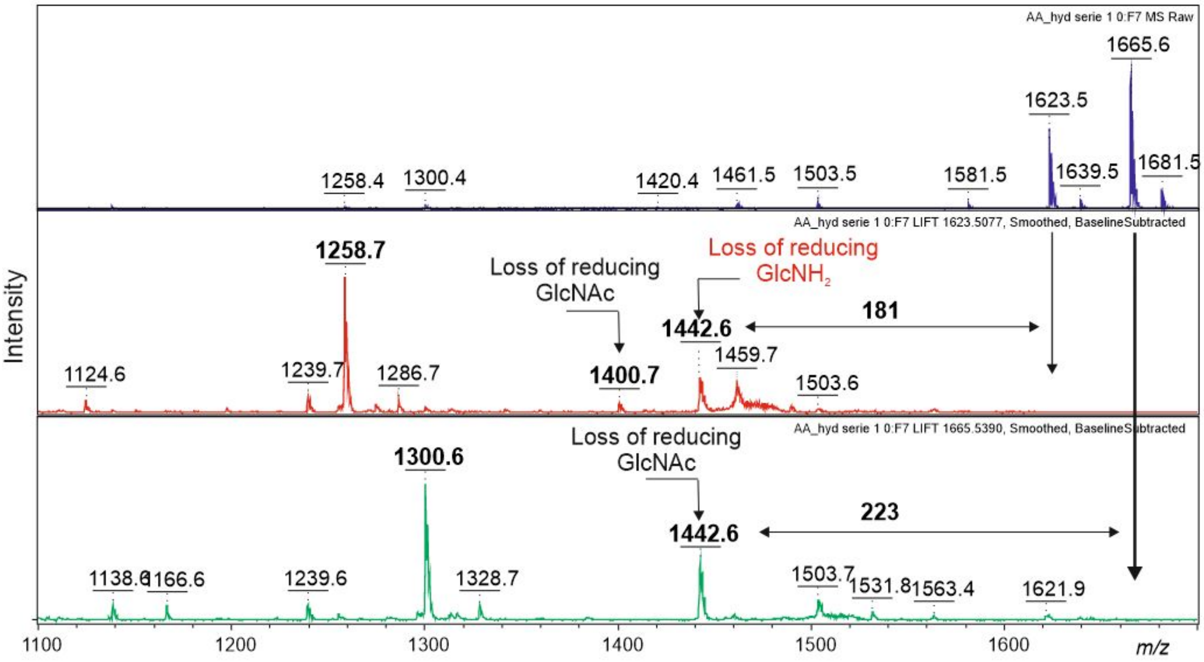

Figure S5: Negative mode CID spectra of a biantennary glycan with one remaining GlcNAc. The upper panel, shows the chromatographic separation on a PGC column of partially de-*N*-acetylated A^4A^4. The extracted ion chromatograms show all five stages of de-*N*-acetylation (intact, -1, -2, -3 and -4 N-acetyl groups). The numbers give the nominal *m/z* of the $[M+2H^+]^{2+}$ mass peaks. The panels below show the lower mass range of the negative mode MS/MS spectra of variants with one remaining *N*-acetyl group per glycan. Differentiation of peaks 1 and 2 relies on the observed much faster de-*N*-acetylation of the reducing end GlcNAc. Peaks 3 and 4 exhibit strong *m/z* = 424 ions indicating the presence of intact GlcNAc in one of the antennae. The allocation of this intact GlcNAc to the 3 or 6-arm was facilitated by consideration of D-type ions, which derive from the 6-arm. [7].

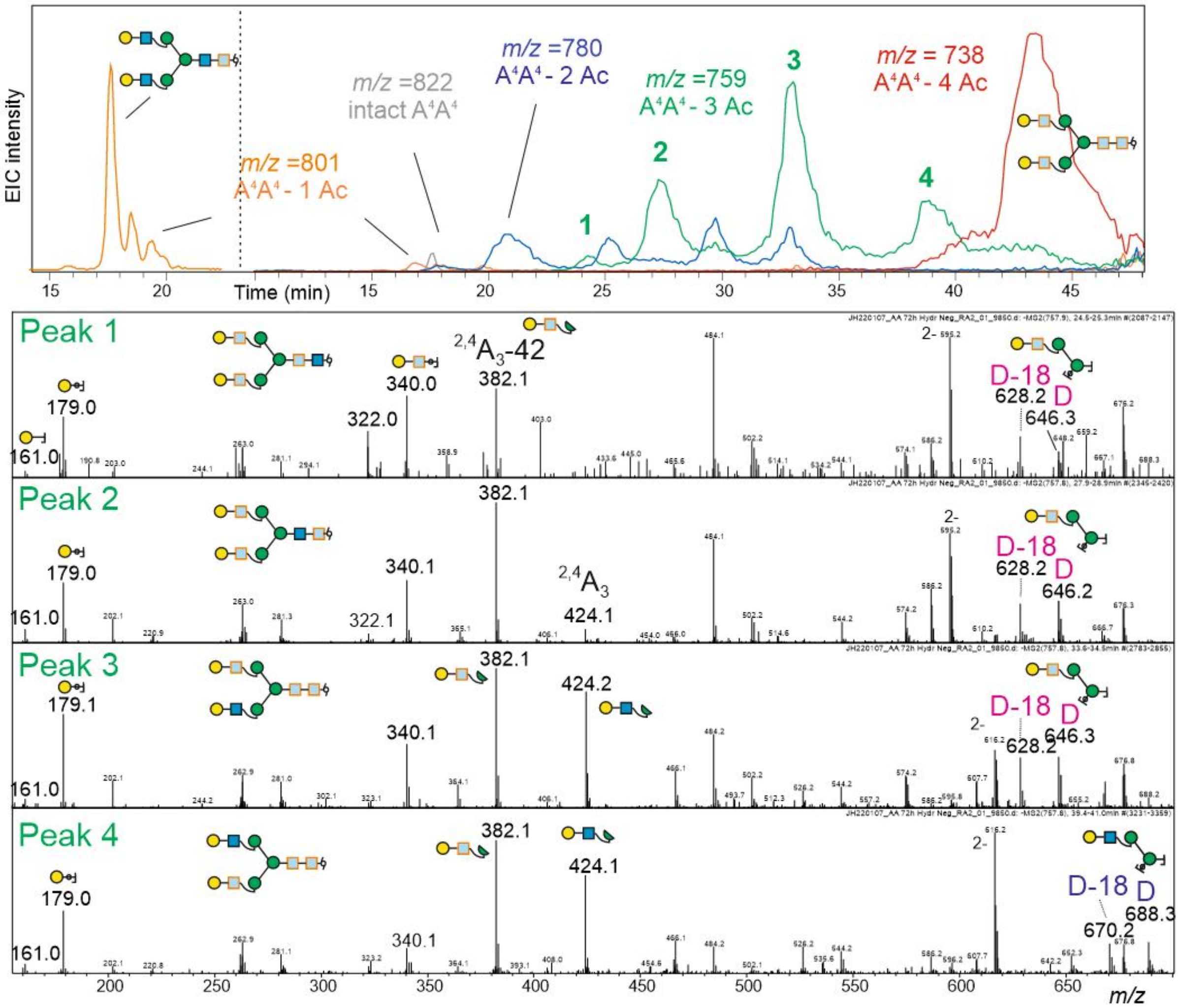

Figure S6. Influence of 3-substitution on susceptibility to hydrazine hydrate. Panel **A**: A mixture of A^4A^4, (AF)(AF) and ^{13}C-galactose labeled A^3A^3 was treated with hydrazine hydrate at 100°C for the times indicated. Aliquots were analyzed by PGC-LC-MS. ^{13}C-containing galactoses are emphasized by bold red circles. In panel **A**, results are depicted as summed spectra, where peak heights are an estimate for the relative abundance of each variant. Panel **B** shows the XIC traces of doubly de-*N*-acetylated N-glycans detected as doubly protonated species $[M+2H^+]^{2+}$ of the 24 h sample. The upper trace shows the variants of A^4A^4, where the large peaks labeled as 1, 3 and, 5) originate from variants that contain a de-*N*-acetylated reduced GlcNAc (i.e. $GlcNH_2$) and the small peaks (labeled with 2, 4, and 6) originate from variants that contain reduced GlcNAc that escaped de-*N*-acetylation. The middle and lower trace show the doubly de-*N*-acetylated structures (AF)(AF) and A^3A^3, respectively.

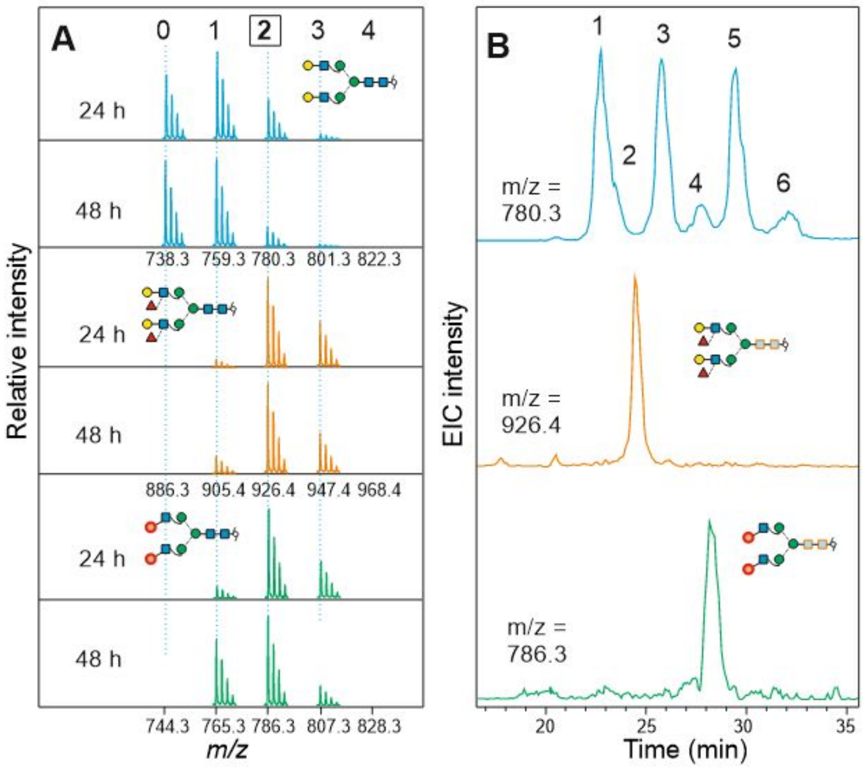

Figure S7. Separation of de-*N*-acetylation variants by cation exchange chromatography. Glycans of different degree of de-N-acetylation were applied to an CIEX cartridge and eluted with a volatile salt gradient. Analysis of peak fractions by MALDI-TOF MS indicated poor separation of charge variants.

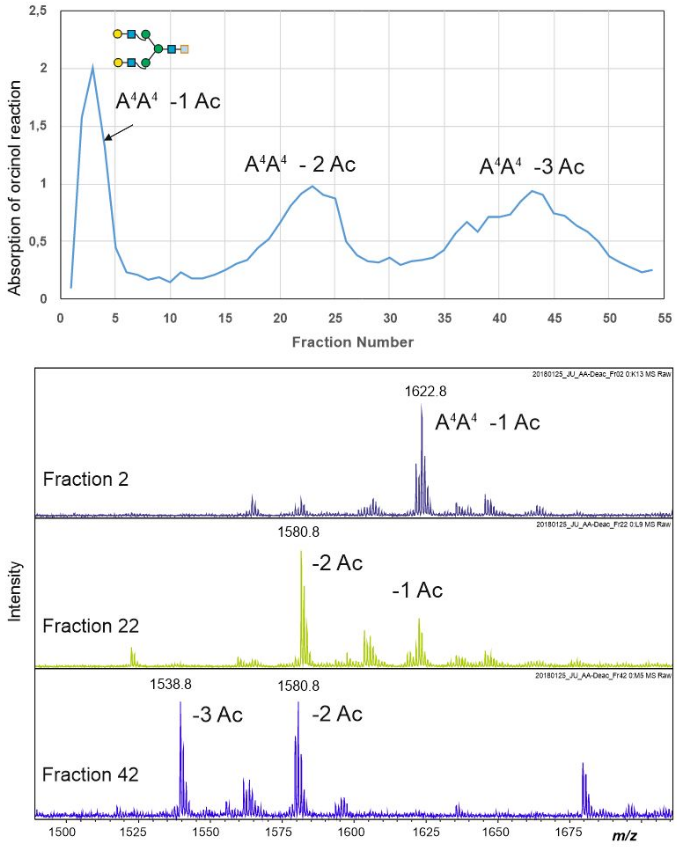

Figure S8. Separation of variously de-*N*-acetylated glycans by HILIC. Glycans (A^4A^4) of different degree of de-*N*-acetylation were applied to a ZIC HILIC column. The effluent was monitored by ESI-MS. Panel **A** shows the extracted ion chromatograms for the different products. Analysis of fractions by MALDI-TOF MS showed perfect separation according to charge (= degree of de-*N*-acetylation) (panel **B**).

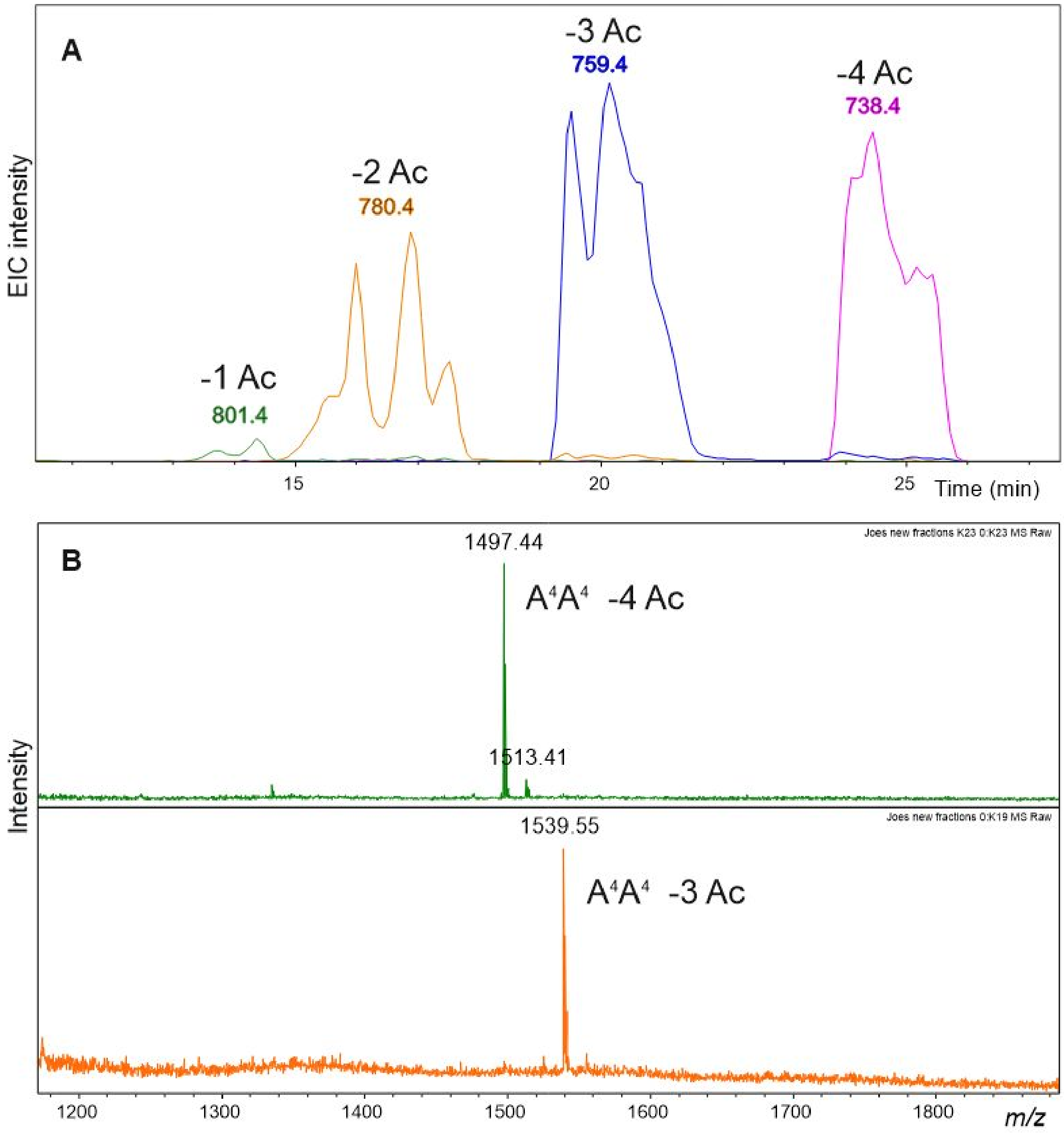

Figure S9. ESI-MS spectra of peaks obtained by amide-HILIC separation of partially de-*N*-acetylated $A^4A^4F^6$ from porcine fibrin. Peaks with m/z = 874.3, 853.3, 832.3 and 811.3 represent glycans lacking 1, 2, 3 and 4 *N*-acetyl groups, respectively.

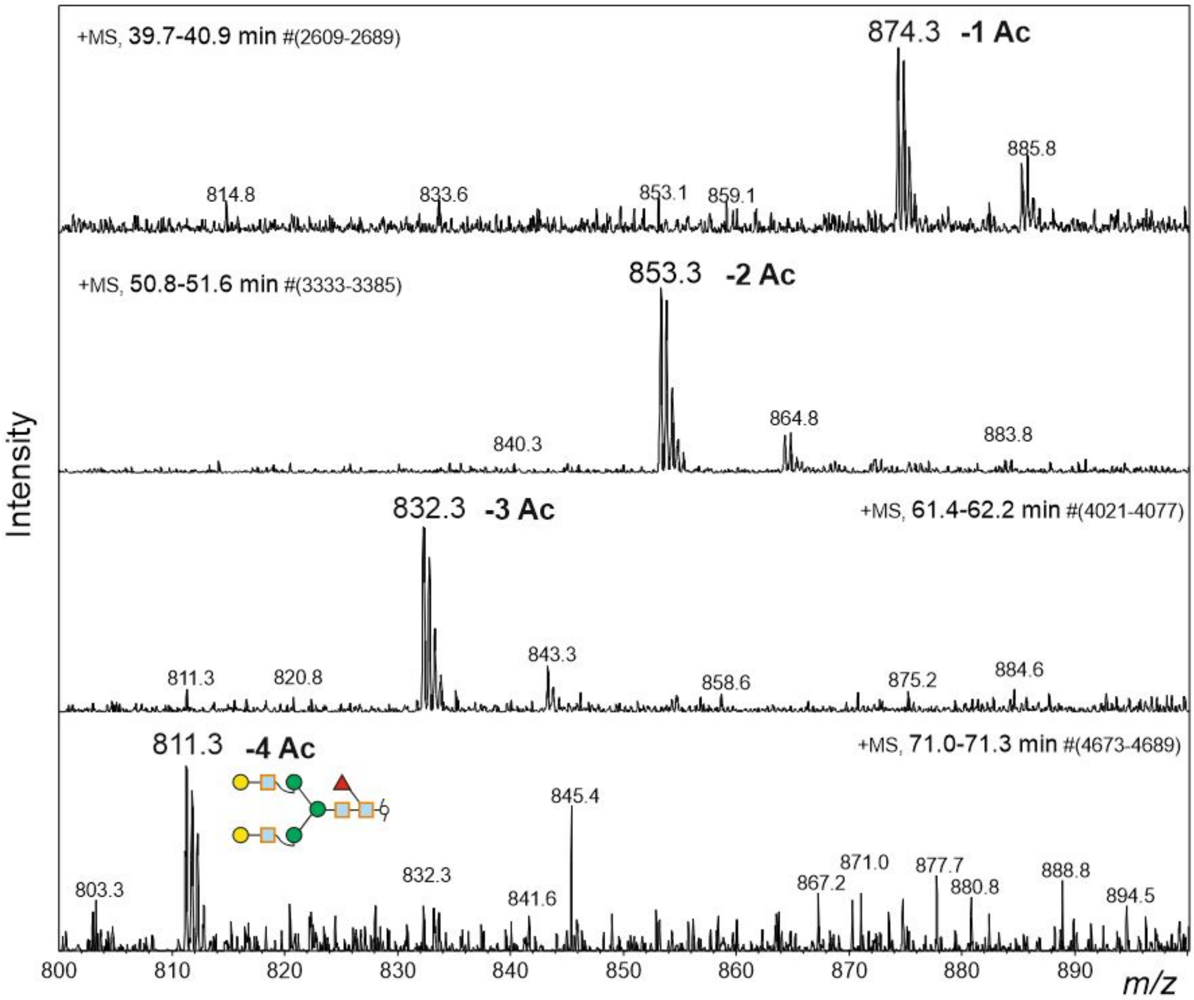

Figure S10. Co-elution of native and of deuterium labeled glycans on PGC. Panel **A**: Verification of co-elution of native and of four-times CD_3-labeled A^4A^4. Panel **B**: MALDI-TOF MS spectrum of the applied sample.

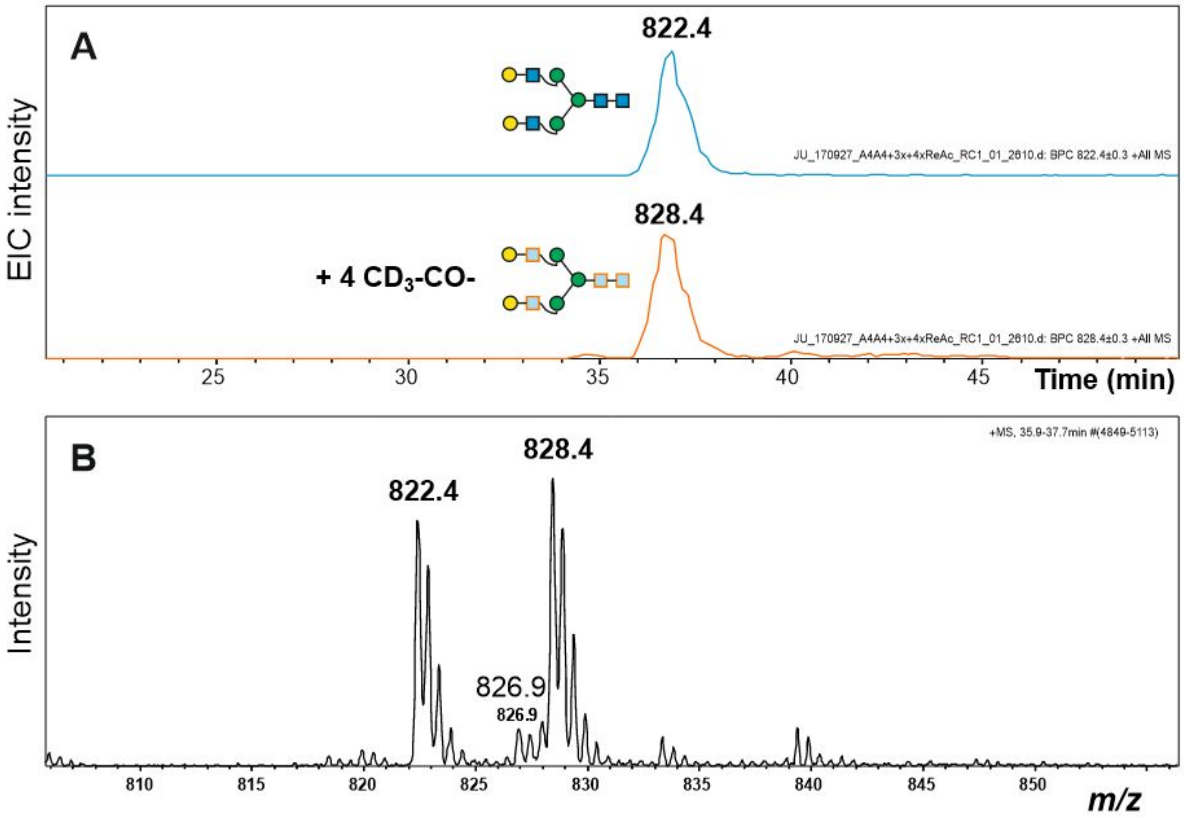

Figure S11. Isotope-coding of tri- and tetra-antennary N-glycans. Reduced N-glycans of recombinant erythropoietin were de-*N*-acetylated for 96 h, subsequently re-*N*-acetylated with $^{13}C_1$-acetic anhydride and subjected to PGC-LC-ESI-MS. The large panel shows the XIC traces for the fully converted tri- and tetra-antennary glycans. The inserts depict the respective sum spectra. "i" denotes peaks arising from a combination of incomplete de-*N*-acetylation and isotopic impurity.

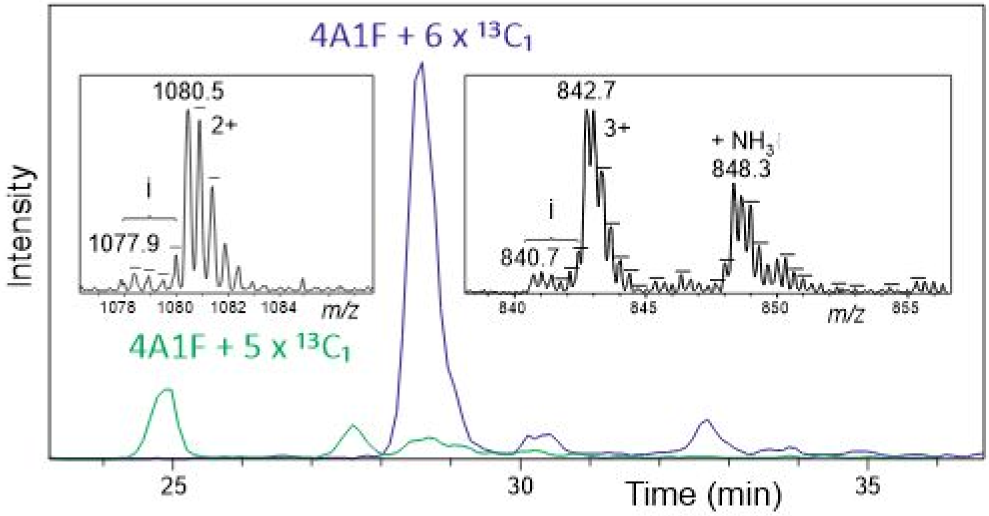

Figure S12. MS/MS spectra of MH+ ions of unlabeled and labeled mucin-type O-glycans. The corresponding MS1 spectra derived by PGC-LC-ESI-MS using an Orbitrap instrument are shown as Figure 5 in the main text.

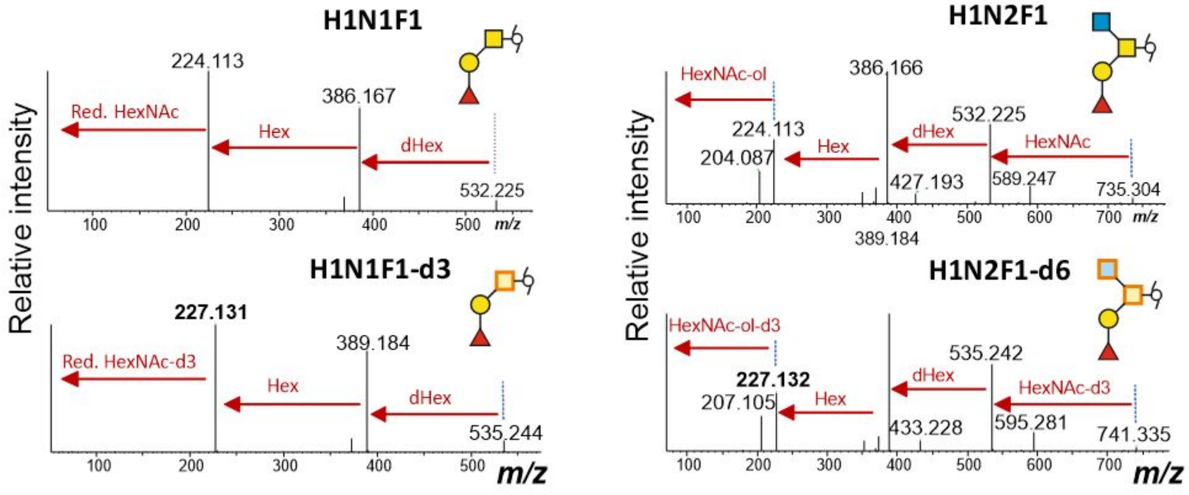

Supporting References:

(1) Grass, J.; Pabst, M.; Kolarich, D.; Poltl, G.; Leonard, R.; Brecker, L.; Altmann, F. Discovery and structural characterization of fucosylated oligomannosidic N-glycans in mushrooms. *J. Biol. Chem.* **2011**, *286* (8), 5977-5984. DOI: 10.1074/jbc.M110.191304.

(2) Helm, J.; Grunwald-Gruber, C.; Thader, A.; Urteil, J.; Fuhrer, J.; Stenitzer, D.; Maresch, D.; Neumann, L.; Pabst, M.; Altmann, F. Bisecting Lewis X in Hybrid-Type N-Glycans of Human Brain Revealed by Deep Structural Glycomics. *Anal. Chem.* **2021**, *93* (45), 15175-15182. DOI: 10.1021/acs.analchem.1c03793.

(3) Pabst, M.; Bondili, J. S.; Stadlmann, J.; Mach, L.; Altmann, F. Mass + retention time = structure: a strategy for the analysis of N-glycans by carbon LC-ESI-MS and its application to fibrin N-glycans. *Anal. Chem.* **2007**, *79* (13), 5051-5057. DOI: 10.1021/ac070363i.

(4) Pabst, M.; Altmann, F. Influence of electrosorption, solvent, temperature, and ion polarity on the performance of LC-ESI-MS using graphitic carbon for acidic oligosaccharides. *Anal Chem* **2008**, *80* (19), 7534-7542. DOI: 10.1021/ac801024r.

(5) Helm, J.; Hirtler, L.; Altmann, F. Towards Mapping of the Human Brain N-Glycome with Standardized Graphitic Carbon Chromatography. *Biomolecules* **2022**, *12* (1), 85. DOI: 10.3390/biom12010085.

(6) Grunwald-Gruber, C.; Thader, A.; Maresch, D.; Dalik, T.; Altmann, F. Determination of true ratios of different N-glycan structures in electrospray ionization mass spectrometry. *Anal. Bioanal. Chem.* **2017**, *409* (10), 2519-2530. DOI: 10.1007/s00216-017-0235-8.

(7) Harvey, D. J.; Crispin, M.; Scanlan, C.; Singer, B. B.; Lucka, L.; Chang, V. T.; Radcliffe, C. M.; Thobhani, S.; Yuen, C. T.; Rudd, P. M. Differentiation between isomeric triantennary N-linked glycans by negative ion tandem mass spectrometry and confirmation of glycans containing galactose attached to the bisecting (beta1-4-GlcNAc) residue in N-glycans from IgG. *Rapid Commun Mass Spectrom* **2008**, *22* (7), 1047-1052. DOI: 10.1002/rcm.3470.
